# Exposure to ambient gaseous pollutant and daily hospitalizations for Sjögren’s syndrome in Hefei: A time-series study

**DOI:** 10.3389/fimmu.2022.1028893

**Published:** 2022-10-27

**Authors:** Tian-Ping Zhang, Li-Jun Wang, Shan Wang, Ping Wang, Xiao-Hui Zhou, Li Wang, Chun-Mei Yang, Xiao-Mei Li

**Affiliations:** ^1^ Department of Rheumatology and Immunology, The First Affiliated Hospital of USTC, Division of Life Sciences and Medicine, University of Science and Technology of China, Hefei, China; ^2^ Department of Infectious Diseases, The First Affiliated Hospital of Anhui Medical University, Hefei, China; ^3^ Department of Rheumatology, The First People’s Hospital of Hefei (Binhu Hospital), Hefei, China; ^4^ Department of Rheumatology, The First People’s Hospital of Hefei, Hefei, China; ^5^ Department of Rheumatology and Immunology, the Third People’s Hospital of Hefei, Hefei, China; ^6^ Department of Scientific Research, The First Affiliated Hospital of USTC, Division of Life Sciences and Medicine, University of Science and Technology of China, Hefei, China

**Keywords:** gaseous pollutants, Sjögren’s syndrome, time-series study, autoimmune disease, air pollutant

## Abstract

**Objective:**

Increasing evidence suggested that gaseous pollutants were associated with the development of autoimmune diseases, while there were few studies on the association between gaseous pollutants and Sjögren’s syndrome (SS). This study sought to assess the relationship between exposure to several gaseous pollutants and the hospitalizations for SS.

**Methods:**

The data regarding SS hospitalizations, gaseous pollutants, and meteorological factors in Hefei from 2016 to 2021 were collected. A distributed lag non-linear model combined with a generalized linear model were adopted to analyze the association between gaseous pollutants and SS hospitalizations, and stratified analyses were also conducted.

**Results:**

We detected significant associations between gaseous pollutants (NO_2_, SO_2_, O_3_, CO) and SS hospitalizations. Exposure to NO_2_ was linked with the elevated risk of hospitalizations for SS (*RR*=1.026, lag1 day). A positive correlation between CO exposure and hospitalizations for SS was found (*RR*=1.144, lag2 day). In contrast, exposure to SO_2_, O_3_ was respectively related to the decreased risk of hospitalizations for SS (SO_2_: *RR*=0.897, lag14 day; O_3_: *RR*=0.992, lag9 day). Stratified analyses found that female patients were more vulnerable to these gaseous pollutants. SS patients ≥ 65 years were more susceptible to NO_2_, CO exposure, and younger patients were more vulnerable to O_3_ exposure. In addition, exposure to O_3_, CO in cold season were more likely to affect hospitalizations for SS.

**Conclusion:**

Our results demonstrated a significant association between exposure to NO_2_, CO and elevated risk of hospitalizations for SS, and SO_2_, O_3_ exposure might be linked to reduced risk of SS hospitalizations.

## Introduction

Sjögren’s syndrome (SS) is a chronic, systemic autoimmune disease characterized by the impaired function of the exocrine glands, which lead to several clinical symptoms such as xerostomia and dry eye (1). Moreover, this disease could affect other systems in the body, primarily included the nervous system, musculoskeletal system, kidneys, skin, lungs, and blood vessels (2). SS could be classified as primary SS (pSS) alone or secondary SS (sSS) according to the presence or absence of other autoimmune diseases, such as systemic lupus erythematosus (SLE), rheumatoid arthritis (RA), systemic sclerosis (SSc) (3). It is generally considered that pSS is the most common autoimmune disease after RA, with a prevalence of 0.1-4.8% among different populations (4). In addition to dry eyes and mouth, most patients with SS also experience pain, fatigue, anxiety, and other symptoms, which could seriously affect people’s physical health (5). At present, the etiology and pathogenesis of SS are not completely clear, and studies have shown that genetic variation and environmental factors greatly affect the occurrence and development of SS (6). It is important to note that the identified genetic risk variants only convey modestly increased risk, further suggesting that other factors were involved in SS development. For example, SS patients often point out the influence of the weather on their symptoms, and believe that wet weather, extreme cold could aggravate the symptoms (7, 8).

More attention had been focused on air pollution because of its harmful effects on health. Some gaseous pollutant, such as carbon monoxide (CO), sulphur dioxide (SO_2_) and nitrogen dioxide (NO_2_), might be involved in regulating many important biological processes, including the increase of free radicals in the body, activation of the immune system and triggering inflammation (9, 10). Previous studies showed that air pollutants could increase the risks of atopic dermatitis, diabetes mellitus, and multiple sclerosis (11–13). In addition, Zhao et al. found that exposure to high levels of NO_2,_ SO_2_ could affect the risk of first admissions for SLE (14). Another study also reported that short-term exposure to NO_2_ and CO had some effect on acute gout hospitalizations (15). These studies suggested that air pollution had some effects on autoimmune disease.

Current studies confirmed a more important role of environmental risk factors in the pathogenesis of SS. For example, one study by Xin et al. (16) suggested that extreme environmental conditions exposure could increase the risk of the outpatient visits for SS. Nevertheless, the role of other environmental factors, such as gaseous pollutants, in the pathogenesis of SS also required further analysis. For all we know, available study on the association between gaseous pollutants and SS were still scarce in humid subtropical regions. We performed this time-series study to investigate the effect of several ambient gaseous pollutants (NO_2_, SO_2_, O_3_, CO) on daily hospitalizations for SS in Hefei City, and identified the susceptible subpopulations and season.

## Material and methods

### Study area and study population

This time-series study was a retrospective, ecological study, and carried out in Hefei (31°52′N, 117°17′E), which was located in the central part of Anhui Province. Hefei featured a subtropical humid monsoon climate with moderate rainfall, and covered a total area of 11,445.1 km^2^ with 9.465 million population by the end of 2021.

The SS hospitalizations of this study were obtained from three hospitals which most SS patients sought medical help from, including the First Affiliated Hospital of University of Science and Technology of China, the First People’s Hospital of Hefei, and the Third People’s Hospital of Hefei, from 1 January 2016 to 31 December 2021 in Hefei. We first obtained the electronic medical records of all hospitalized patients from the hospital information system (HIS) of these hospitals, and identified SS patients based on their diagnosis in the electronic medical records. Moreover, all SS patients were diagnosed by senior rheumatologists according to the 2002 American European Consensus Group (AECG) classification criteria (17) and the following information of SS patients was collected, including age, gender, residential address, and date of hospitalization. Meanwhile, the SS patients who lived outside Hefei, had incomplete demographic information, and were hospitalized in other departments other than the department of rheumatology and immunology were excluded from this study. The ethics approval was granted by the Ethics Committee of The First Affiliated Hospital of University of Science and Technology of China (2022-RE-037).

### Pollutants and meteorological data

In this study, we used the air pollutants concentration of the residential address of SS patient to reflect their amount of exposure to air pollutants. Air pollution data (24-h for NO_2_, SO_2_, CO, and 8-h for O3) from 2016 to 2021 were collected through the environmental monitoring network (including 10 air quality monitoring stations) established by Hefei Environmental Monitoring Center. The daily air pollutant data was an average of 10 monitoring stations. Data on daily relative humidity (RH, %) and mean temperature (°C) during this study period were obtained from the China Meteorological Data Service Center (http://data.cma.cn/).

### Statistical analysis

The effect of gaseous pollutants on hospitalizations for SS was investigated with a distributed lag non-linear model (DLNM) combined with a generalized linear model in this study. These two models were respectively used to describe the additional lag-response correlation and the traditional exposure-response correlation. Since the daily admissions for SS was considered as a small probability event, thus, the DLNM with a quasi-Poisson distribution was adopted to investigate the association between gaseous pollutant and SS hospitalizations (15). Spearman analysis was used to analyze the correlation between each covariate, and two variables whose correlation coefficient less than 0.7 could not enter in the same model for avoiding multicollinearity. Finally, the model for NO_2_, SO_2_, CO, O_3_ were shown as below:

Y_t_ ~ quasipossion(μ_t_)

Log(μ_t1_)_NO2_=α_1_+β_1_NO_2t,*l*_+ns(SO_2_,3)+ns(RH,3)+ns(time,6*6)+η_1_DOW_t_+γ_1_Holiday_t_

Log(μ_t2_)_SO2_=α_2_+β_2_SO_2t,*l*_+ns(NO_2_,3)+ns(MT,3)+ns(RH,3)+ns(time,8*6)+η_2_DOW_t_+γ_2_Holiday_t_

Log(μ_t3_)_O3_=α_3_+β_3_O_3t,*l*_+ns(SO_2_,4)+ns(MT,4)+ns(RH,4)+ns(time,6*6)+η_3_DOW_t_+γ_3_Holiday_t_

Log(μ_t4_)_CO_=α_4_+β_4_CO_t,*l*_+ns(O_3_,3)+ns(RH,3)+ns(time,7*6)+η_4_DOW_t_+γ_4_Holiday_t_

The subscript *t* referred to the day of observation, *Yt* and μ*t* were the actual and expected SS hospitalizations on day *t*, respectively. In above models, α represented the intercept of the model. In NO_2_ model, *NO_2t,l_* was the dlnm cross basis matrix of *NO_2t_*, *l* represented the lag day, *β_1_* was the vector of *NO_2t_*, ns() means natural cubic spline. A natural cubic spline curve of time with 6 *dfs*/year was adopted to control the seasonality and long-term trend (18). DOW was the dummy variable of the day of week and the two-category variable *Holiday_t_* was used to control the effect of holidays. Quasi-Poisson Akaike Information Criterion (Q-AIC) was preferred to identify the optimal *dfs* and determined the final model parameters. In order to identify the susceptible populations, further stratified analyses were conducted according to different age (<65 years vs ≥65 years), gender (male vs female). In addition, the relationship between gaseous pollutants and SS hospitalizations was respectively analyzed among hot season (April-September) and cold season (October-March). The statistical analyses were conducted using R software version 3.6.1 (http://www.R-project.org) with “dlnm” and “splines” packages, and *P <*0.05 (two-sided) was considered as statistically significant.

### Sensitivity analyses

Sensitivity analyses were performed by varying the *dfs* in the ns function for gaseous pollutants (3-5 *dfs*), meteorological variables (3-5 *dfs*) and time (6-8 *dfs* per year) in turn in the model to verify the robustness of our models.

## Results

### Descriptive analysis

**Figure 1:**
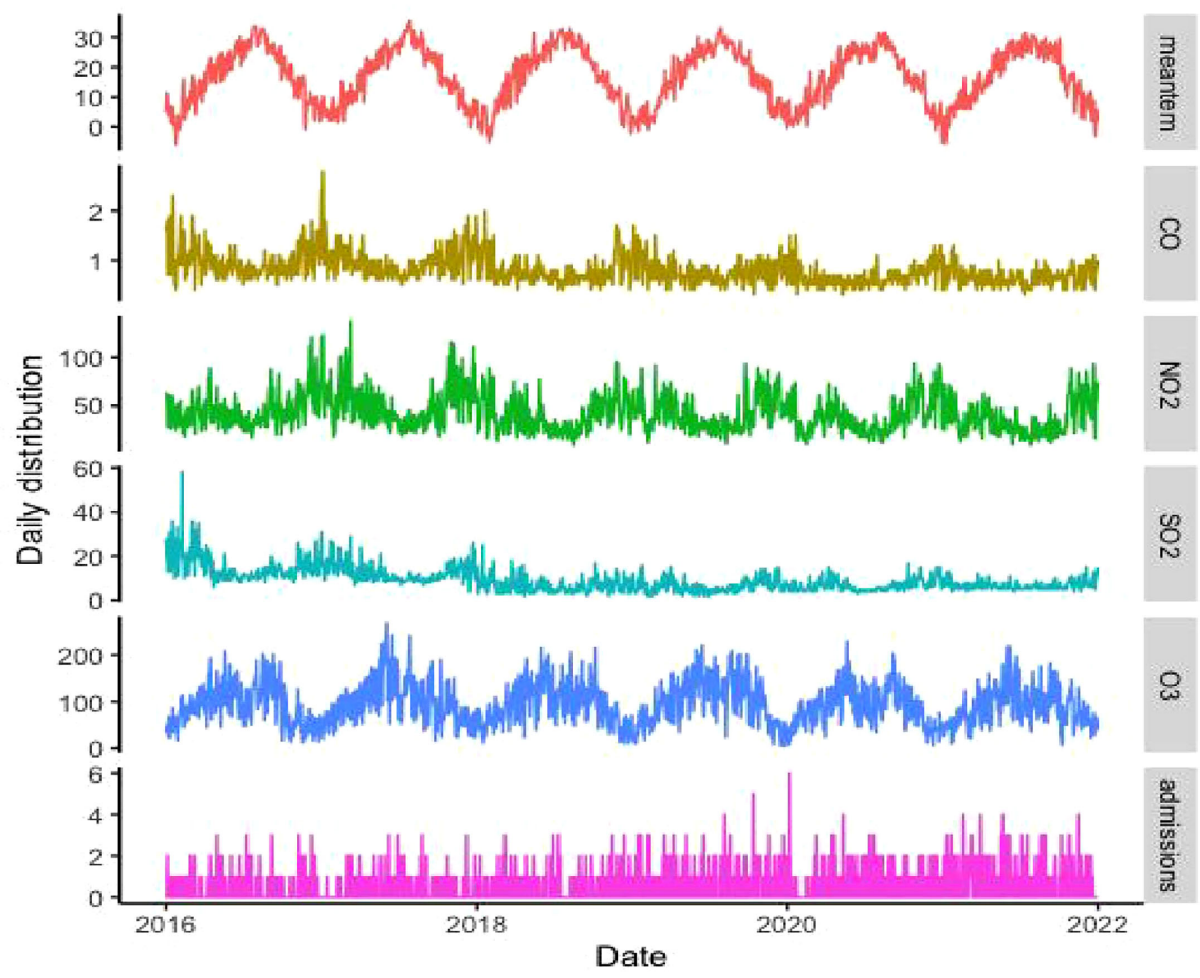
The time series of gaseous pollutants, mean temperature, and hospitalizations for SS from 2016 to 2021 in Hefei.

A total of 1119 SS hospitalizations were collected in Hefei from 1 January 2016 to 31 December 2021, and the characteristics of these SS patients, ambient gaseous pollutant and meteorological factors were shown in **Table 1**. Among the SS patients, 1061 cases were female (94.8%), 350 cases (31.3%) were aged 65 years or older, and there were more hospital admissions for SS in hot season (580, 51.8%). The daily mean values for NO_2_, CO, SO_2_, and O_3_ (24-h for NO_2_, CO, SO_2_ and 8-h for O_3_) were 42.09 μg/m^3^ (range: 9 μg/m^3^ – 137 μg/m^3^), 0.80 mg/m^3^ (range: 0.3 mg/m^3^-2.8 mg/m^3^), 8.97 μg/m^3^ (range: 2 μg/m^3^ – 58 μg/m^3^) and 94.57 μg/m^3^ (4 μg/m^3^ –269 μg/m^3^). The number of daily hospital admissions for SS ranged from 0 to 6 during the study period. **Figure 1** presented the temporal trends of gaseous pollutants, mean temperature, and hospitalizations for SS from 2016 to 2021 in Hefei. The results of Spearman rank correlation analysis and scatter plot were arranged in **Figure S1**, and the correlation coefficients between any two variables are less than 0.7.

**Table 1:** The basic information on SS admission, meteorological variables and gaseous pollutants in Hefei City from 2016 to 2021.

Variables	N	Mean (SD)	Min	*P_25_*	*P_50_*	*P_75_*	*P_90_*	Max
Admissions	1119	0.51(0.77)	0	0	0	1	2	6
Male	58	0.03(0.16)	0	0	0	0	0	1
Female	1061	0.48(0.75)	0	0	0	1	1	6
Age < 65 years	769	0.35(0.63)	0	0	0	1	1	4
Age ≥ 65 years	350	0.16(0.41)	0	0	0	0	1	4
Hot season	580	0.26(0.61)	0	0	0	0	1	4
Cold season	539	0.25(0.60)	0	0	0	0	1	6
Mean temperature, °C	–	16.77(9.26)	-5.9	8.7	17.2	24.7	28.3	35.6
Relative humidity, %	–	76.99(12.26)	33	69	78	86	93	99
NO_2_, μg/m^3^	–	42.09(18.90)	9	28	38	54	68.7	137
CO, mg/m^3^	–	0.80(0.28)	0.3	0.6	0.7	0.9	1.2	2.8
SO_2_, μg/m^3^	–	8.97(5.07)	2	5	8	11	15	58
O_3_, μg/m^3^	–	94.57(44.54)	4	60	90	125	156	269

### The association between gaseous pollutants and SS hospitalizations

#### Overall effects

**Figure 2:**
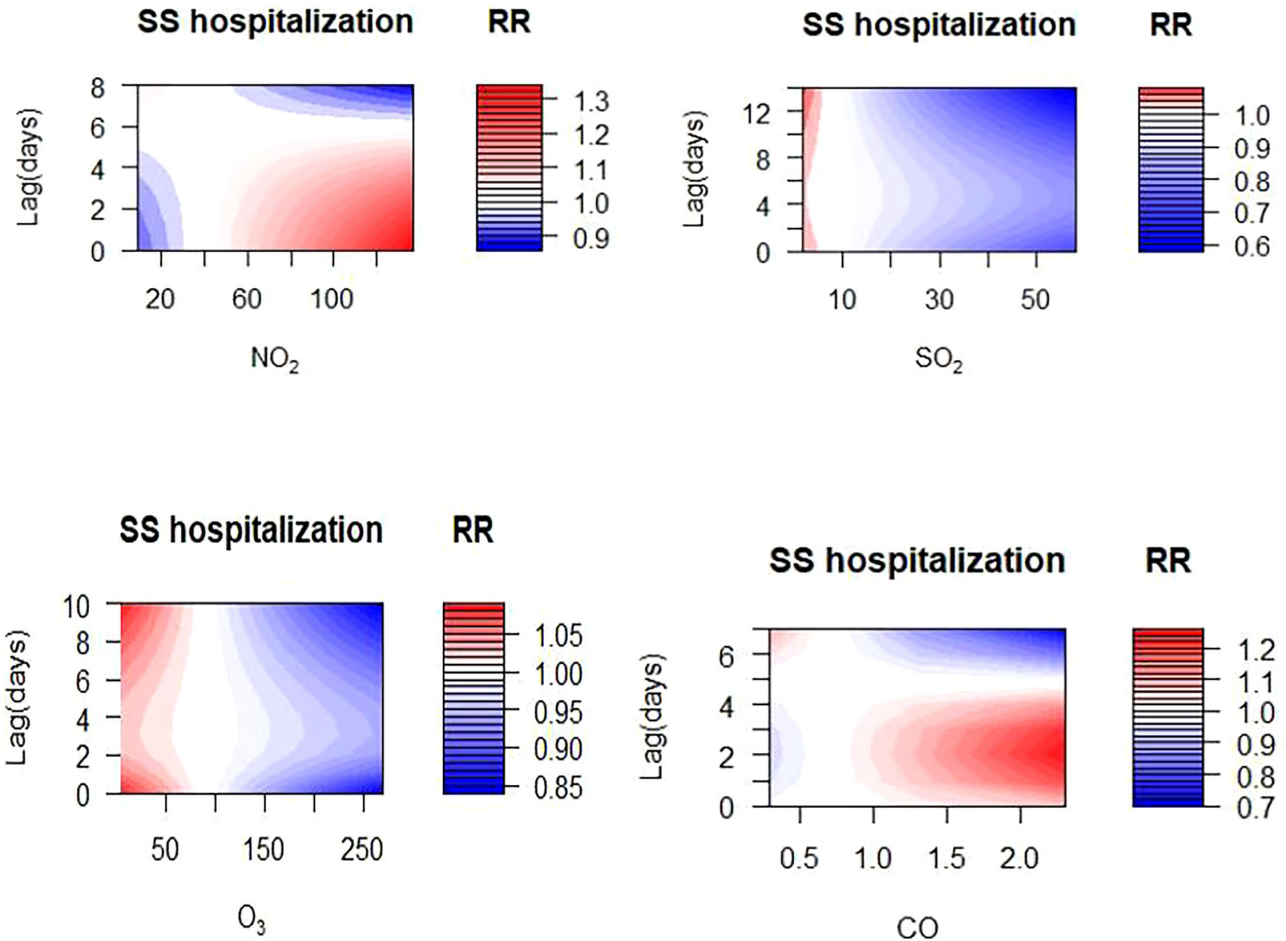
Contour plots for relative risk of hospitalizations for SS admissions along NO_2_, SO_2_, O_3_ and CO at lag periods in Hefei.

The exposure-response relationships between SS hospitalizations and gaseous pollutants (NO_2_, SO_2,_ O_3,_ and CO) in different lag days were presented in **Figure 2**. The results indicated a meaningful positive correlation between high concentrations of NO_2_ and CO (reference concentrations of 38 μg/m^3^ and 0.7 mg/m^3^, respectively) and the elevated risk of SS hospitalizations, while exposure to high concentration of SO_2_ and O_3_ (reference concentrations of 8 μg/m^3^ and 90 μg/m^3^, respectively) could reduce the risk of SS hospitalization. In addition, the concentration-response relationships between NO_2_, SO_2_, O_3_, CO and SS hospitalizations were shown in **Figure S2**.

#### Effects of NO_2_ on SS hospitalizations

**Figure 3:**
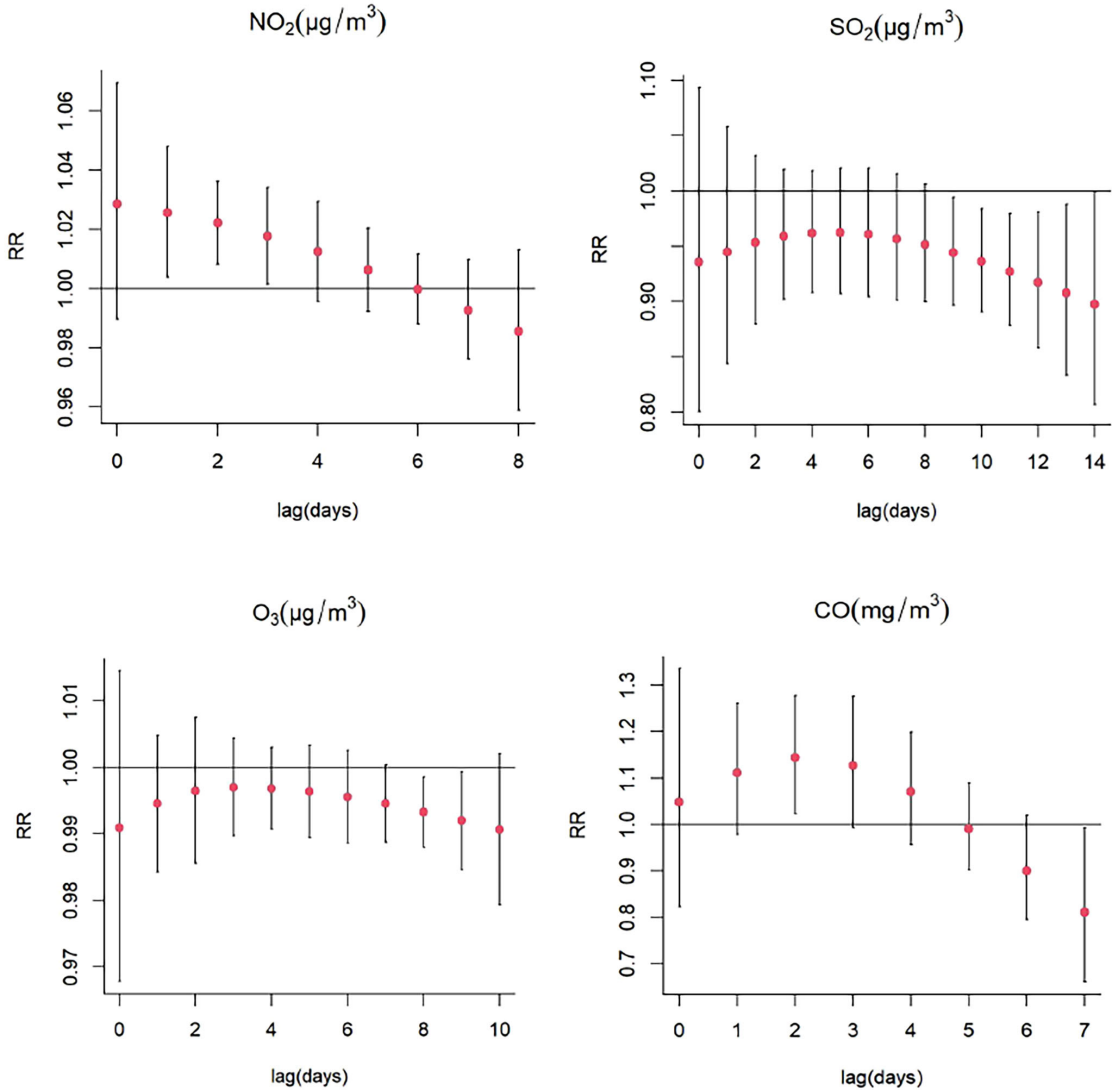
Lag-specific relative risks (%) in hospitalizations for SS per 10 (or 1)-unit increase in daily mean concentrations of gaseous pollutants in the model.

**Figure 4:**
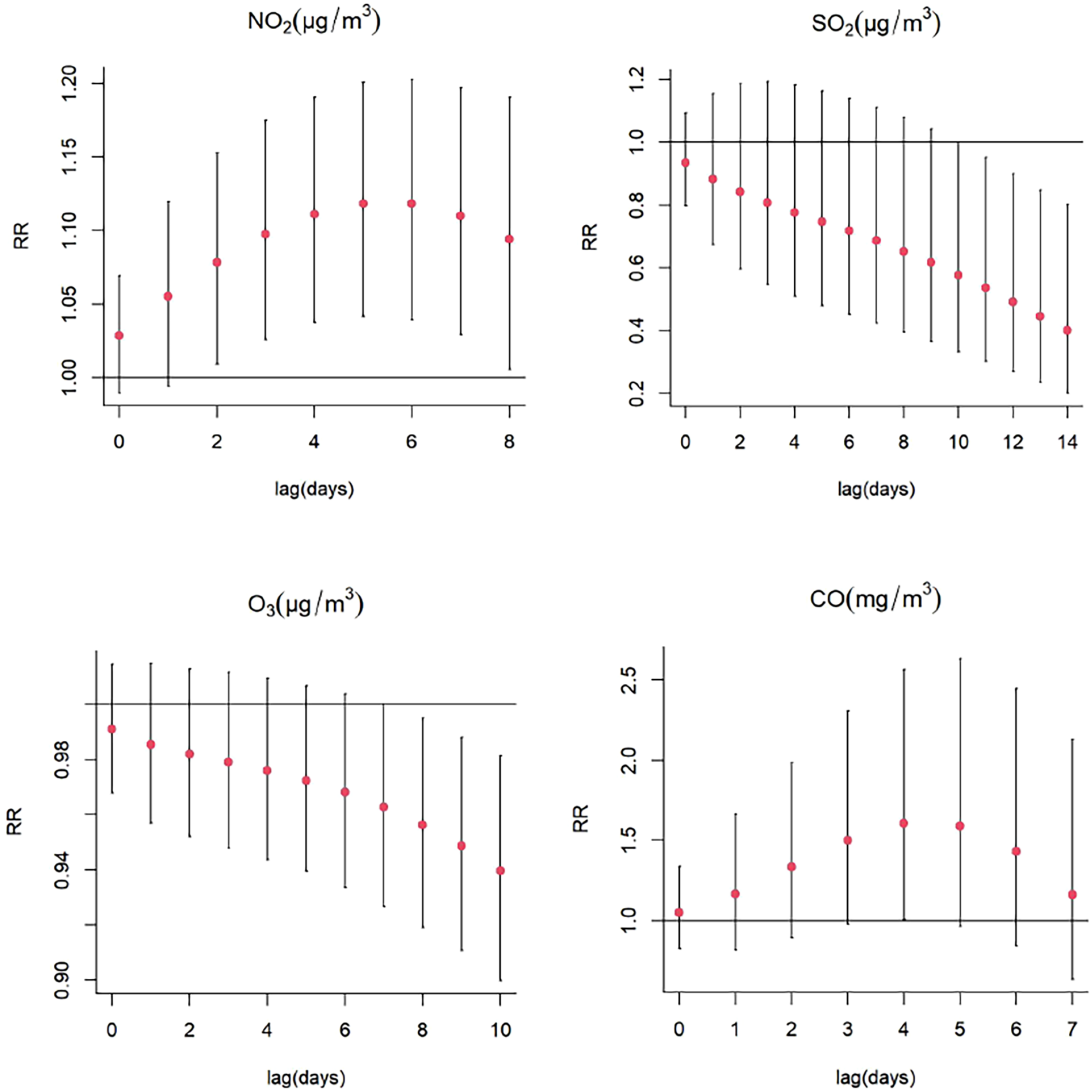
Cumulative risks (%) in hospitalizations for SS per 10 (or 1)-unit increase in daily mean concentrations of gaseous pollutants in the model.

**Figure 5:**
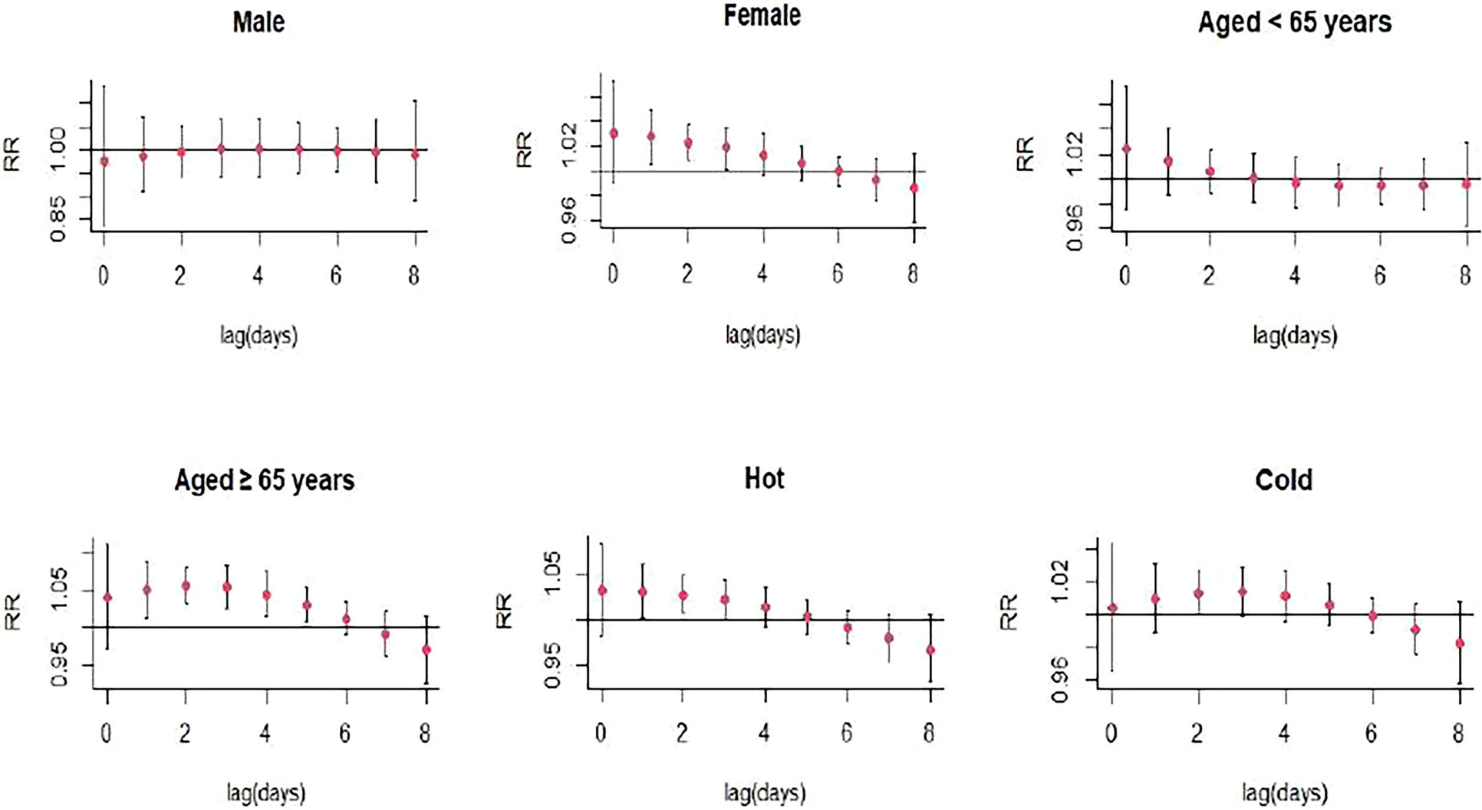
Lag-specific relative risks (95% *CI*) of SS hospitalizations per 10 unit increase in the daily concentrations of NO_2_ in models stratified by age, gender, and season.

There was a significant single-day effect between NO_2_ and SS hospitalizations with every 10 μg/m^3^ increase in NO_2_ concentration from lag1 (RR: 1.026, 95% CI: 1.004-1.048) to lag3 (RR:1.018, 95% CI: 1.002-1.034), and the highest RR of SS admissions was at lag1 (**Figure 3**; **Table S1**). Moreover, the meaningful estimated risk effects were found to from lag0-2 (RR: 1.079, 95% CI: 1.009-1.153) to lag0-8 (RR:1.094, 95% CI: 1.005-1.191) in the cumulative lag structure, and the highest RR was found at lag5 (RR: 1.118, 95% CI: 1.041-1.201) (**Figure 4**; **Table S1**). When stratified by gender, age, season, the results suggested and NO_2_ exposure was positively correlated with the number of SS hospitalizations in female (RR:1.027, 95% CI: 1.005-1.050, lag1), the patients ≥65 years (RR: 1.055, 95% CI: 1.031-1.080, lag2), hot seasons (RR:1.031, 95% CI: 1.003-1.060, lag1), and cold seasons (RR: 1.014, 95% CI: 1.000-1.027, lag2) (**Figure 5**; **Table S2**).

#### Effects of SO_2_ on SS hospitalizations

**Figure 6:**
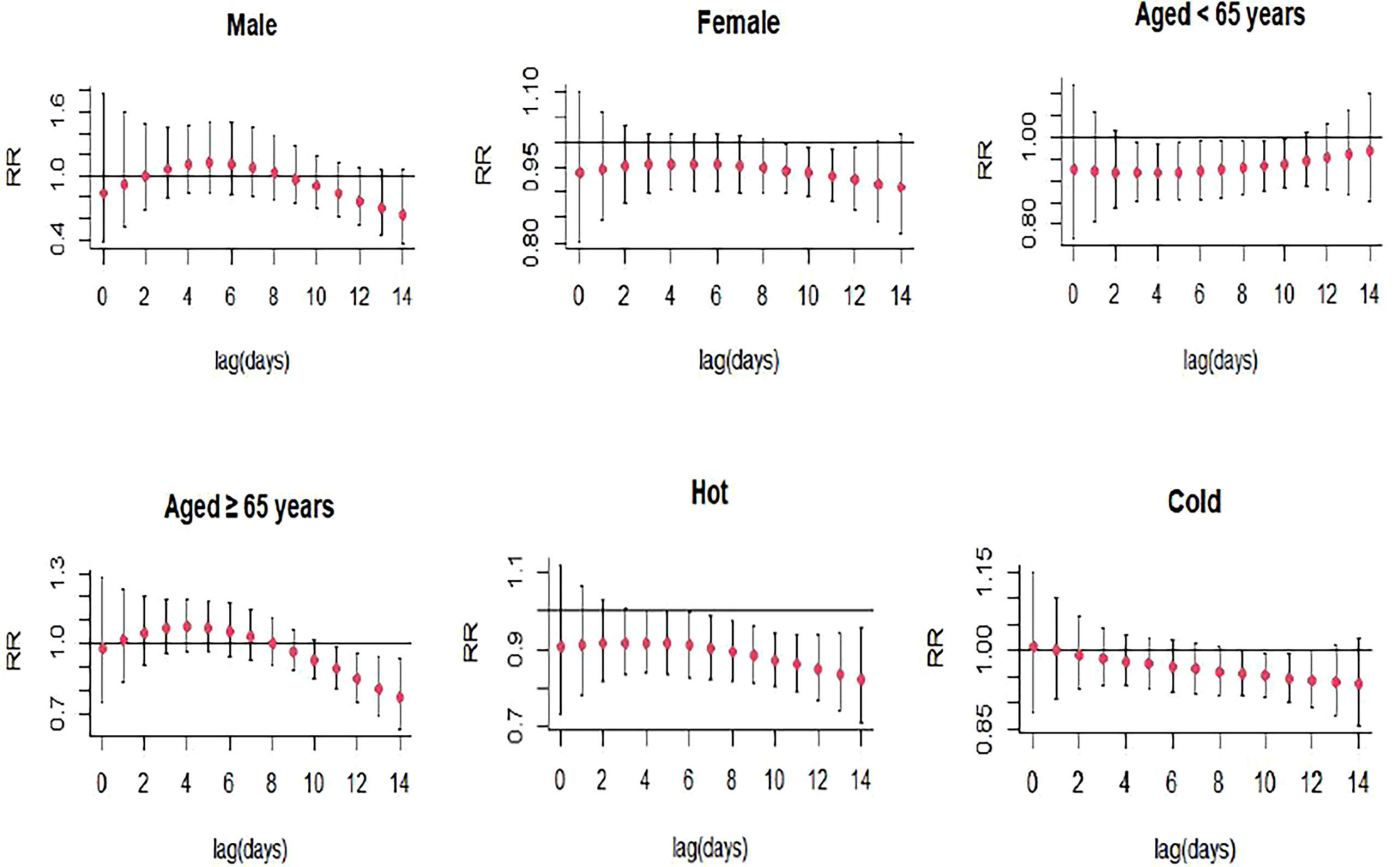
Lag-specific relative risks (95% *CI*) of SS hospitalizations per 10 unit increase in the daily concentrations of SO_2_ in models stratified by age, gender, and season.

For every 10 μg/m^3^ increase in SO_2_ concentration, the association between SO_2_ and the daily hospitalizations for SS was statistically significant from lag9 (RR: 0.944, 95% CI: 0.896-0.994) to lag14 (RR:0.897, 95% CI: 0.807-0.998) in the single-day lag structure, and the lowest RR was found at lag14 (**Figure 3**; **Table S3**). In multi-day cumulative effect, the risk of SS hospitalization decreased with increasing SO_2_ over the lag0-11 (RR:0.536, 95% CI: 0.302-0.952) to lag0-14 (RR:0.400 95% CI: 0.199-0.803), and the lowest RR was also at lag0-14 (**Figure 4**; **Table S3**). In addition, the effects of SO_2_ exposure on SS hospitalizations were statistically significant in female (RR: 0.925, 95% CI: 0.864-0.991, lag12), the patients < 65 years (RR: 0.917, 95% CI: 0.852-0.983, lag4), the patients ≥65 years (RR: 0.771, 95% CI: 0.636-0.934, lag14), hot seasons (RR: 0.824, 95% CI: 0.711-0.955, lag14), and cold seasons (RR: 0.947, 95% CI: 0.902-0.994, lag11) (**Figure 6**; **Table S4**).

#### Effects of O_3_ on SS hospitalizations

**Figure 7:**
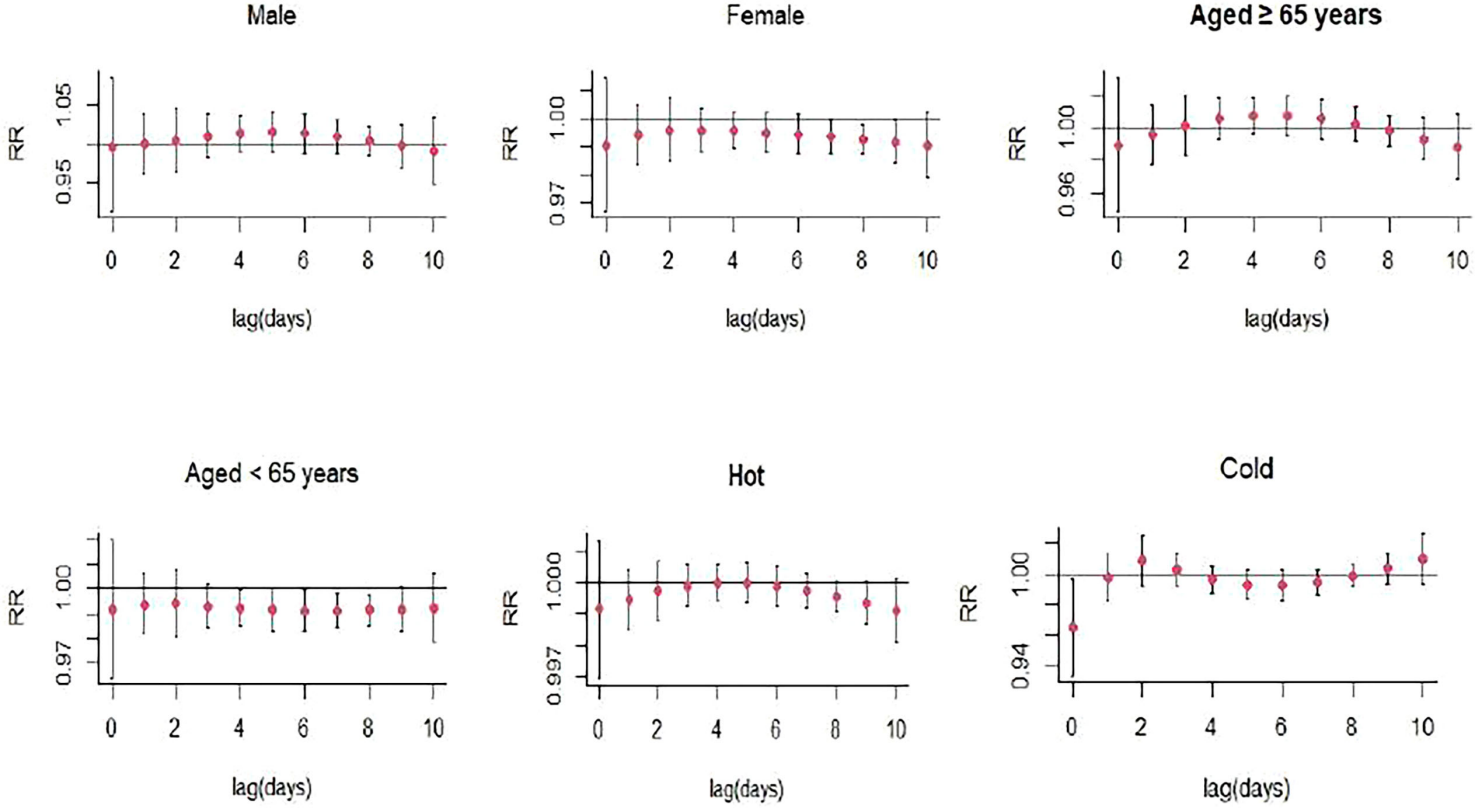
Lag-specific relative risks (95% *CI*) of SS hospitalizations per 10 unit increase in the daily concentrations of O_3_ in models stratified by age, gender, and season.

An inverse single-day single day lag association was found between O_3_ and the risk of SS hospitalizations (lag8, RR: 0.993, 95% CI: 0.988-0.999, and lag9, RR: 0.992, 95% CI: 0.985-0.999, per 10 μg/m^3^ increase in O_3_ concentration) (**Figure 3**; **Table S5**). In the cumulative lag structure, the estimated risk effects decrease from lag0-8 (RR: 0.956, 95% CI: 0.919-0.995) to lag0-10 (RR: 0.940, 95% CI: 0.900-0.981) (**Figure 4**; **Table S5**). The results of the subgroup analyses also revealed that the relation between O_3_ exposure and a decreased risk of SS hospitalizations was consistently significant in female (RR: 0.992, 95% CI: 0.984-0.999, lag9), the patients < 65 years (RR: 0.991, 95% CI: 0.984-0.998), lag7), and cold seasons (RR: 0.965, 95% CI: 0.934-0.997, lag1) (**Figure 7**; **Table S6**).

#### Effects of CO on SS hospitalizations

**Figure 8:**
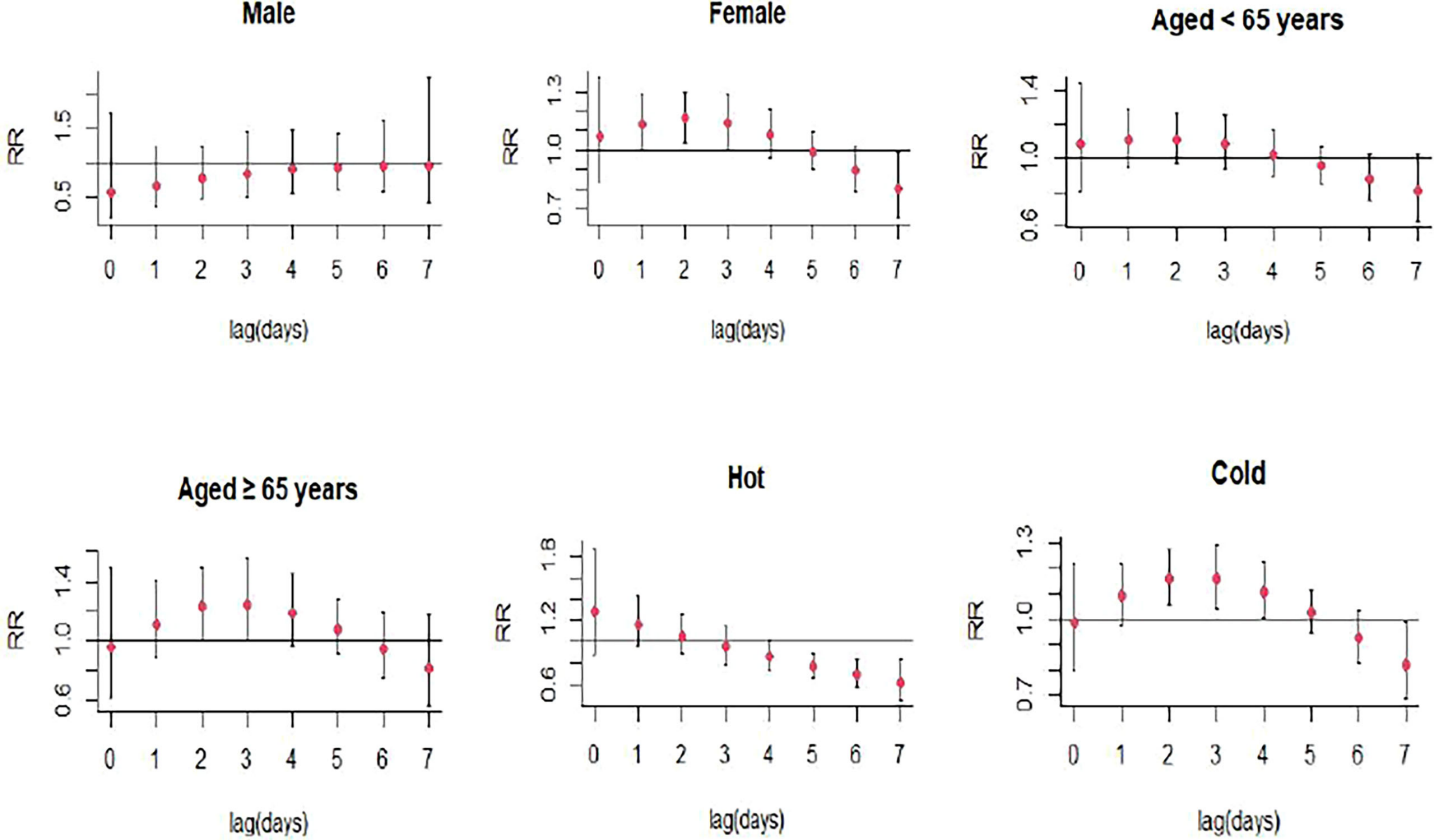
Lag-specific relative risks (95% *CI*) of SS hospitalizations per 1 unit increase in the daily concentrations of CO in models stratified by age, gender, and season.

The single-day lag association between CO and SS hospitalizations was statistically significant at lag2 (RR: 1.144, 95% CI: 1.023-1.278) with every 1 mg/m^3^ increase in CO concentration (**Figure 3**; **Table S7**), and the multi-day cumulative risk was significant at lag0-4 (RR: 1.607, 95% CI: 1.006-2.566) (**Figure 4**; **Table S7**). Interestingly, this study also observed a significant relationship between CO and an increased SS hospitalizations in female (RR: 1.160, 95% CI: 1.035-1.299, lag2), the patients ≥ 65 years (RR: 1.227, 95% CI: 1.007-1.496, lag2), and cold seasons (RR: 1.160, 95% CI: 1.040-1.293, lag3) (**Figure 8**; **Table S8**).

### Sensitivity analyses

The sensitivity analyses results suggested that the effects of gaseous pollutants on SS hospitalizations were stable under *df* variations for time, gaseous pollutants, meteorological factors. Hence, these models were considered to be reliable and robust **(** **Figures S3–S6** **)**.

## Discussions

Air pollution, including gaseous pollutants, had become an important public problem threatening human health. Several studies had suggested that there was a close association between exposure to gaseous pollutants and the risk of a variety of autoimmune diseases (14, 15, 19), which provided important clues for further research on the pathogenesis of autoimmune diseases. The results of a recent study showed that cold, damp and long sunshine duration might associated with the number of SS patients visiting hospitals, and the effects were influenced by age, sex (16). This suggested that the role of environmental factors in the pathogenesis of SS deserved more attention. In this study, we identified significant associations between exposure to gaseous pollutants and the hospitalizations for SS. Exposure to NO_2_, CO was positively related to the risk of hospitalizations for SS, while exposure to SO_2_, O_3_ was negatively related to the risk of hospitalizations for SS.

As common gaseous pollutants produced by traffic, exposure to high concentrations of NO_2_, CO might cause various adverse health effects. One cohort study showed that the average exposure levels of O_2_, CO in 5-year prior to RA diagnosis were significantly associated with the high incidence of RA (20). A time-series study by Wu et al. found that exposure to high-concentration NO_2_ was significantly related to an increased risk of RA readmissions (21). Severe dryness of the mucosal surface in eyes and oral cavity were the primary clinical manifestation among SS patients, and the patients with SS who were exposed to air pollution was associated with more severe abnormalities of the ocular surface and eye irritation (22). In addition, environmental NO_2_ concentration was proved to be associated with dry eye syndrome (23). This might support our results that exposure to NO_2_ was associated with an increased risk of daily hospitalizations for SS. Moreover, we found a promoting effect of CO exposure on the risk of hospitalization for SS. This effect could also be observed in another autoimmune disease, where adults exposed to CO for one year had a higher risk of developing RA in a population-based cohort study (24). At present, the biological mechanism by which NO_2_ and CO affect the risk of SS remained unclear. It was possible that exposure to these pollutants might cause oxidative stress and increase the release of proinflammatory mediators within the lung and systemically. Chronic inflammation of the lungs might promote the susceptibility to diseases characterized by inflammation, which was considered a suitable biomarker of exposure to relevant gaseous pollutants, and might contribute to the occurrence of rheumatic diseases (20). We hypothesized that exposure to high concentrations of CO and NO_2_ might be involved in some inflammatory processes and promoted the onset of inflammation in SS patients.

Among the gaseous pollutants, the lower level of O_3_ and high concentrations of nitrogen oxides often appeared in proximity to high-traffic areas (25). Several studies had suggested significant associations between O_3_ exposure and the risk of human diseases. One previous study found that O_3_ exposure could reduce the risk of interstitial lung disease in pSS patients (26). In this study, our results showed the protective effect of O_3_ exposure against the risk of SS hospitalizations. Exposure to SO_2_ were also negatively related to the risk of SS hospitalizations in the present study. Similarly, the study by Jung et al. found that exposure to increased O_3_ and SO_2_ were negatively associated with the increased risk of SLE (27). These results suggested that SO_2_ and O_3_ exposures had certain protective effect on the risk of medical visits for autoimmune diseases included SS, while the specific mechanism was still unclear due to the lack of relevant studies. Intriguingly, O_3_ was involved in the high Th2 response in airway cells by enhancing the type 2 innate lymphoid cell (ILC)-related pathways (28). Therefore, the O_3_-associated expansion of the Th2 pathway through ILCs might partially explain the potential protective effect of O_3_ on SS development by ameliorating Th17/Th1-related signaling pathways. In addition, ground-level O_3_ was a secondary pollutant produced by photochemical reactions between traffic-related air pollutants, including NO_2_ and volatile organic compounds (26). Our results also implied that the primary pollutants (NO_2_ and CO) had greater importance on the SS hospitalizations in comparison to the secondary pollutants (O_3_) produced by photochemical processes.

The stratified analyses showed that the effects of NO_2_, SO_2_, O_3_, and CO exposure on SS hospitalizations remained significant in female SS patients but not in male patients. Women tend to exhibit higher immune reactivity, with different numbers or reactivity of cells constituting the immune responses and different resistance to target organ damage, which might result in different effects of air pollutants on the risk of autoimmune diseases among male and female patients (29). NO_2_, CO exposure were associated with higher risk of SS hospitalizations in elderly patients, while O_3_ exposure was linked to lower risk of SS hospitalizations in young patients. This might be caused by aging leading to the alterations in the function of Toll like receptors (30), which were potent inflammation mediators in SS and played an important function in salivary tissue. We also found a statistically significant association between CO exposure and the risk of SS hospitalizations during the cold season. This might be associated with the dry environment in cold season, and exposure to dry environment could result in the deterioration of tear function unit in SS patients with dry eye symptom through promoting inflammatory activity (31).

There were several strengths in this study. First, as we all know, this was the first time-series study to examine the association between exposure to gaseous pollutants and hospitalizations for SS. Second, stratified analyses by gender, age and season were conducted to identify the populations and seasons that were particularly vulnerable to gaseous pollutants. In addition, we acknowledged that some limitations existed in this study. First, we only collected SS hospitalizations data from three hospitals in Hefei, which might not be able to represent SS inpatients in Hefei. Another limitation was that the design of this study had an ecological fallacy, which might restrict the ability of our study to explore the causal relationships. Finally, the association between SS activity, complications and gaseous pollutants was not analyzed because of missing data. In order to analyze the precise mechanisms responsible for the associations between gaseous pollutants and SS, further studies based on epidemiological investigations and functional investigations should be conducted.

In summary, our study provided key evidence that exposure to NO_2_, CO was positively related to the increased risk of hospitalizations for SS in Hefei. While, a negative association was identified between exposure to SO_2_, O_3_ and SS hospitalizations. Stratified analyses found that the effect of these gaseous pollutants on risk of SS hospitalizations could be modified by gender. The effect of NO_2_, CO, O_3_ exposure on SS hospitalizations was affected by age, and the relationship between CO, O_3_ exposure and SS hospitalizations remained significant in cold season. This study was of great significance for further explore the role of gaseous pollutants in the pathogenesis of SS.

## Data availability statement

The raw data supporting the conclusions of this article will be made available by the authors, without undue reservation.

## Ethics statement

This study was approved by the Ethical Committee of the First Affiliated Hospital of USTC (Hefei, Anhui, China).

## Author contributions

X-ML, C-MY and T-PZ designed the study. SW, PW, X-HZ and LW participated in the data collection. L-JW performed data processing. T-PZ conducted the data analysis and drafted the manuscript. X-ML and C-MY contributed to manuscript revision. All authors contributed to the article and approved the submitted version.

## Acknowledgments

This work was supported by the National Natural Science Foundation of China (U21A20365).

## Conflict of interest

The authors declare that the research was conducted in the absence of any commercial or financial relationships that could be construed as a potential conflict of interest.

## Publisher’s note

All claims expressed in this article are solely those of the authors and do not necessarily represent those of their affiliated organizations, or those of the publisher, the editors and the reviewers. Any product that may be evaluated in this article, or claim that may be made by its manufacturer, is not guaranteed or endorsed by the publisher.
