# Supplementary material for: Exposure to ambient gaseous pollutant and daily hospitalizations for Sjögren’s syndrome in Hefei: A time-series study
Source: Front Immunol. 2022 Oct 27;13:1028893. doi: 10.3389/fimmu.2022.1028893 (PMC9646840; doi:10.3389/fimmu.2022.1028893)
Supplement: Supplementary file 1 [file DataSheet_1.doc]

**Table S1** The association between NO_2_ exposure and SS hospitalizations in Hefei from 2016 to 2021

| Single-day | *RR* (95% *CI*) | Multi-day | *RR* (95% *CI*) |
| --- | --- | --- | --- |
| 0 | 1.028(0.988-1.070) | 0-0 | 1.028(0.989-1.070) |
| 1 | 1.026(1.004-1.048) ^a^ | 0-1 | 1.055(0.994-1.120) |
| 2 | 1.022(1.008-1.037) ^a^ | 0-2 | 1.079(1.009-1.153) ^a^ |
| 3 | 1.018(1.002-1.034) ^a^ | 0-3 | 1.098(1.025-1.175) ^a^ |
| 4 | 1.012(0.996-1.029) | 0-4 | 1.111(1.037-1.191) ^a^ |
| 5 | 1.006(0.993-1.020) | 0-5 | 1.118(1.041-1.201) ^a^ |
| 6 | 1.000(0.988-1.012) | 0-6 | 1.118(1.039-1.203) ^a^ |
| 7 | 0.993(0.976-1.010) | 0-7 | 1.110(1.029-1.197) ^a^ |
| 8 | 0.986(0.959-1.013) | 0-8 | 1.094(1.005-1.191) ^a^ |

RR: Relative risk; CI: confidence interval; a:*P* < 0.05.

**Table S2** The association between NO_2_ exposure and SS hospitalizations in different subgroups (*RR*, 95% *CI*)

| lag | Male | Female | Age < 65 years | Age ≥ 65 years | Hot season | Cold season |
| --- | --- | --- | --- | --- | --- | --- |
| 0 | 0.977(0.839-1.138) | 1.031(0.989-1.073) | 1.024(0.975-1.075) | 1.038(0.970-1.111) | 1.032(0.983-1.084) | 1.003(0.965-1.043) |
| 1 | 0.988(0.910-1.072) | 1.027(1.005-1.050) ^a^ | 1.014(0.988-1.041) | 1.049(1.012-1.088) ^a^ | 1.031(1.003-1.060) ^a^ | 1.010(0.989-1.031) |
| 2 | 0.996(0.943-1.052) | 1.023(1.008-1.038) ^a^ | 1.006(0.989-1.024) | 1.055(1.031-1.080) ^a^ | 1.028(1.007-1.048) ^a^ | 1.014(1.000-1.027) ^a^ |
| 3 | 1.001(0.940-1.066) | 1.018(1.001-1.035) ^a^ | 1.001(0.981-1.021) | 1.053(1.025-1.082) ^a^ | 1.022(1.000-1.044) ^a^ | 1.014(0.999-1.029) |
| 4 | 1.003(0.941-1.069) | 1.013(0.996-1.030) | 0.997(0.977-1.017) | 1.044(1.016-1.073) ^a^ | 1.014(0.992-1.036) | 1.011(0.996-1.027) |
| 5 | 1.002(0.950-1.057) | 1.006(0.992-1.021) | 0.995(0.979-1.012) | 1.029(1.006-1.054) ^a^ | 1.004(0.985-1.023) | 1.006(0.993-1.019) |
| 6 | 0.999(0.954-1.048) | 1.000(0.988-1.012) | 0.995(0.981-1.009) | 1.011(0.991-1.032) | 0.992(0.974-1.011) | 0.999(0.988-1.010) |
| 7 | 0.996(0.931-1.065) | 0.993(0.976-1.010) | 0.995(0.975-1.016) | 0.990(0.962-1.020) | 0.980(0.956-1.005) | 0.991(0.976-1.006) |
| 8 | 0.991(0.890-1.103) | 0.985(0.958-1.013) | 0.996(0.963-1.030) | 0.969(0.925-1.015) | 0.968(0.933-1.005) | 0.982(0.958-1.008) |
| 0-0 | 0.977(0.839-1.138) | 1.031(0.989-1.073) | 1.024(0.975-1.075) | 1.038(0.970-1.111) | 1.032(0.983-1.084) | 1.003(0.965-1.043) |
| 0-1 | 0.965(0.766-1.215) | 1.059(0.995-1.126) | 1.039(0.965-1.118) | 1.089(0.983-1.206) | 1.064(0.987-1.148) | 1.013(0.955-1.074) |
| 0-2 | 0.961(0.742-1.243) | 1.083(1.012-1.160) ^a^ | 1.045(0.963-1.134) | 1.149(1.026-1.288) ^a^ | 1.094(1.003-1.193) ^a^ | 1.027(0.962-1.096) |
| 0-3 | 0.962(0.737-1.255) | 1.103(1.029-1.183) ^a^ | 1.036(0.962-1.127) | 1.210(1.078-1.359) ^a^ | 1.118(1.019-1.227) ^a^ | 1.041(0.975-1.112) |
| 0-4 | 0.965(0.733-1.269) | 1.117(1.041-1.199) ^a^ | 1.042(0.957-1.135) | 1.263(1.123-1.421) ^a^ | 1.133(1.026-1.251) ^a^ | 1.053(0.985-1.125) |
| 0-5 | 0.967(0.726-1.287) | 1.125(1.045-1.210) ^a^ | 1.037(0.951-1.132) | 1.301(1.152-1.468) ^a^ | 1.127(1.023-1.265) ^a^ | 1.059(0.990-1.134) |
| 0-6 | 0.966(0.717-1.303) | 1.124(1.043-1.212) ^a^ | 1.032(0.944-1.129) | 1.315(1.161-1.490) ^a^ | 1.129(1.007-1.264) ^a^ | 1.059(0.988-1.134) |
| 0-7 | 0.962(0.701-1.320) | 1.116(1.033-1.206) ^a^ | 1.027(0.936-1.126) | 1.302(1.143-1.485) ^a^ | 1.106(0.978-1.251) | 1.049(0.977-1.127) |
| 0-8 | 0.953(0.667-1.363) | 1.100(1.008-1.200) ^a^ | 1.022(0.922-1.133) | 1.262(1.088-1.463) ^a^ | 1.071(0.931-1.232) | 1.031(0.951-1.116) |

*RR:* Relative risk; *CI:* confidence interval; a:*P* < 0.05.

**Table S3** The association between SO_2_ exposure and SS hospitalizations in Hefei from 2016 to 2021

| Single-day | *RR* (95% *CI*) | Multi-day | *RR* (95% *CI*) |
| --- | --- | --- | --- |
| 0 | 0.935(0.800-1.093) | 0-0 | 0.935(0.800-1.093) |
| 1 | 0.945(0.844-1.058) | 0-1 | 0.884(0.676-1.156) |
| 2 | 0.953(0.880-1.032) | 0-2 | 0.842(0.597-1.188) |
| 3 | 0.959(0.902-1.020) | 0-3 | 0.808(0.546-1.194) |
| 4 | 0.962(0.908-1.018) | 0-4 | 0.777(0.509-1.184) |
| 5 | 0.962(0.907-1.020) | 0-5 | 0.747(0.480-1.165) |
| 6 | 0.961(0.904-1.021) | 0-6 | 0.718(0.452-1.140) |
| 7 | 0.957(0.902-1.015) | 0-7 | 0.687(0.424-1.112) |
| 8 | 0.951(0.900-1.006) | 0-8 | 0.653(0.395-1.081) |
| 9 | 0.944(0.896-0.994) ^a^ | 0-9 | 0.617(0.365-1.044) |
| 10 | 0.936(0.891-0.984) ^a^ | 0-10 | 0.578(0.334-1.001) |
| 11 | 0.927(0.978-0.979) ^a^ | 0-11 | 0.536(0.302-0.952) ^a^ |
| 12 | 0.918(0.858-0.981) ^a^ | 0-12 | 0.492(0.269-0.900) ^a^ |
| 13 | 0.908(0.833-0.988) ^a^ | 0-13 | 0.446(0.234-0.849) ^a^ |
| 14 | 0.897(0.807-0.998) ^a^ | 0-14 | 0.400(0.199-0.803) ^a^ |

*RR*: Relative risk; *CI*: confidence interval; a:*P* < 0.05.

**Table S4** The association between SO_2_ exposure and SS hospitalizations in different subgroups (*RR*, 95% *CI*)

| lag | Male | Female | Age < 65 years | Age ≥ 65 years | Hot season | Cold season |
| --- | --- | --- | --- | --- | --- | --- |
| 0 | 0.825(0.385-1.771) | 0.940(0.802-1.101) | 0.927(0.767-1.120) | 0.983(0.750-1.289) | 0.906(0.734-1.117) | 1.007(0.882-1.149) |
| 1 | 0.914(0.522-1.599) | 0.946(0.843-1.061) | 0.923(0.804-1.059) | 1.016(0.835-1.238) | 0.911(0.780-1.064) | 1.000(0.906-1.102) |
| 2 | 0.999(0.670-1.490) | 0.952(0.877-1.032) | 0.920(0.834-1.013) | 1.045(0.909-1.202) | 0.916(0.818-1.025) | 0.992(0.925-1.064) |
| 3 | 1.066(0.780-1.456) | 0.955(0.897-1.017) | 0.918(0.852-0.989) ^a^ | 1.064(0.955-1.186) | 0.918(0.837-1.006) | 0.985(0.933-1.041) |
| 4 | 1.106(0.827-1.479) | 0.957(0.903-1.014) | 0.917(0.856-0.983) ^a^ | 1.071(0.968-1.184) | 0.917(0.840-1.002) | 0.979(0.931-1.029) |
| 5 | 1.120(0.831-1.511) | 0.957(0.902-1.017) | 0.918(0.855-0.986) ^a^ | 1.067(0.962-1.183) | 0.914(0.835-1.001) | 0.974(0.925-1.024) |
| 6 | 1.110(0.816-1.510) | 0.956(0.899-1.017) | 0.921(0.856-0.991) ^a^ | 1.053(0.947-1.171) | 0.909(0.828-0.998) ^a^ | 0.968(0.919-1.020) |
| 7 | 1.078(0.794-1.464) | 0.953(0.897-1.013) | 0.924(0.860-0.993) ^a^ | 1.031(0.929-1.144) | 0.902(0.823-0.989) ^a^ | 0.963(0.916-1.014) |
| 8 | 1.029(0.767-1.380) | 0.949(0.897-1.005) | 0.928(0.868-0.993) ^a^ | 1.002(0.909-1.106) | 0.894(0.819-0.975) ^a^ | 0.959-0.914-1.006) |
| 9 | 0.968(0.733-1.279) | 0.944(0.896-0.995) ^a^ | 0.933(0.877-.0993) ^a^ | 0.969(0.884-1.061) | 0.884(0.814-0.959) ^a^ | 0.955(0.912-0.999) ^a^ |
| 10 | 0.899(0.683-1.185) | 0.939(0.892-0.988) ^a^ | 0.939(0.885-0.997) ^a^ | 0.931(0.851-1.019) | 0.873(0.806-0.945) ^a^ | 0.950(0.909-0.994) ^a^ |
| 11 | 0.827(0.613-1.116) | 0.932(0.881-0.986) ^a^ | 0.946(0.886-1.009) | 0.891(0.807-0.985) ^a^ | 0.861(0.791-0.936) ^a^ | 0.947(0.902-0.994) ^a^ |
| 12 | 0.756(0.530-1.078) | 0.925(0.864-0.991) ^a^ | 0.953(0.880-1.031) | 0.851(0.753-0.961) ^a^ | 0.849(0.769-0.936) ^a^ | 0.943(0.889-1.000) |
| 13 | 0.687(0.444-1.063) | 0.918(0.842-1.001) | 0.960(0.868-1.062) | 0.810(0.695-0.945) ^a^ | 0.836(0.741-0.943) ^a^ | 0.939(0.874-1.010) |
| 14 | 0.622(0.364-1.063) | 0.911(0.818-1.015) | 0.967(0.852-1.098) | 0.771(0.636-0.934) ^a^ | 0.824(0.711-0.955) ^a^ | 0.936(0.856-1.023) |
| 0-0 | 0.825(0.385-1.771) | 0.940(0.802-1.101) | 0.927(0.767-1.120) | 0.983(0.750-1.289) | 0.906(0.734-1.117) | 1.007(0.882-1.149) |
| 0-1 | 0.754(0.202-2.820) | 0.889(0.676-1.168) | 0.856(0.618-1.184) | 0.999(0.627-1.593) | 0.825(0.574-1.187) | 1.006(0.800-1.265) |
| 0-2 | 0.753(0.138-4.104) | 0.846(0.597-1.199) | 0.787(0.519-1.193) | 1.045(0.575-1.898) | 0.756(0.473-1.208) | 0.998(0.742-1.341) |
| 0-3 | 0.803(0.115-5.592) | 0.808(0.543-1.202) | 0.722(0.449-1.160) | 1.111(0.562-2.197) | 0.693(0.404-1.191) | 0.983(0.700-1.381) |
| 0-4 | 0.888(0.108-7.287) | 0.774(0.504-1.187) | 0.663(0.397-1.105) | 1.190(0.570-2.486) | 0.636(0.352-1.149) | 0.962(0.665-1.393) |
| 0-5 | 0.995(0.107-9.225) | 0.741(0.472-1.162) | 0.609(0.356-1.042) | 1.270(0.584-2.759) | 0.581(0.309-1.095) | 0.937(0.633-1.387) |
| 0-6 | 1.104(0.107-11.420) | 0.708(0.443-1.133) | 0.560(0.320-0.981) ^a^ | 1.337(0.594-3.008) | 0.529(0.270-1.036) | 0.907(0.600-1.371) |
| 0-7 | 1.191(0.103-13.780) | 0.675(0.413-1.102) | 0.518(0.289-0.928) ^a^ | 1.379(0.592-3.213) | 0.477(0.234-0.974) ^a^ | 0.874(0.567-1.348) |
| 0-8 | 1.225(0.094-16.020) | 0.641(0.384-1.069) | 0.481(0.261-0.884) ^a^ | 1.382(0.571-3.343) | 0.426(0.200-0.909) ^a^ | 0.838(0.531-1.322) |
| 0-9 | 1.186(0.080-17.674) | 0.605(0.355-1.033) ^a^ | 0.449(0.237-0.848) ^a^ | 1.339(0.532-3.369) | 0.377(0.169-0.840) ^a^ | 0.800(0.495-1.292) |
| 0-10 | 1.067(0.062-18.259) | 0.568(0.325-0.993) ^a^ | 0.421(0.216-0.820) ^a^ | 1.246(0.475-3.267) | 0.329(0.141-0.766) ^a^ | 0.760(0.459-1.259) |
| 0-11 | 0.883(0.044-17.554) | 0.530(0.295-0.950) ^a^ | 0.398(0.198-0.800) ^a^ | 1.111(0.405-3.043) | 0.283(0.116-0.690) ^a^ | 0.720(0.423-1.225) |
| 0-12 | 0.667(0.028-15.768) | 0.490(0.265-0.907) ^a^ | 0.380(0.182-0.790) ^a^ | 0.945(0.327-2.728) | 0.240(0.094-0.615) ^a^ | 0.679(0.387-1.191) |
| 0-13 | 0.458(0.016-13.434) | 0.450(0.234-0.866) ^a^ | 0.364(0.167-0.794) ^a^ | 0.766(0.248-2.367) | 0.201(0.074-0.544) ^a^ | 0.637(0.349-1.163) |
| 0-14 | 0.285(0.007-11.115) | 0.410(0.202-0.833) ^a^ | 0.352(0.152-0.818) ^a^ | 0.590(0.173-2.009) | 0.165(0.057-0.482) ^a^ | 0.596(0.311-1.144) |

*RR:* Relative risk; *CI:* confidence interval; a:*P* < 0.05.

**Table S5** The association between O_3_ exposure and SS hospitalizations in Hefei from 2016 to 2021

| Single-day | *RR* (95% *CI*) | Multi-day | *RR* (95% *CI*) |
| --- | --- | --- | --- |
| 0 | 0.991(0.968-1.014) | 0-0 | 0.991(0.968-1.014) |
| 1 | 0.995(.0984-1.005) | 0-1 | 0.985(0.957-1.015) |
| 2 | 0.996(0.986-1.008) | 0-2 | 0.982(0.952-1.013) |
| 3 | 0.997(0.990-1.004) | 0-3 | 0.979(0.948-1.011) |
| 4 | 0.997(0.991-1.003) | 0-4 | 0.976(0.944-1.009) |
| 5 | 0.996(0.989-1.003) | 0-5 | 0.972(0.939-1.007) |
| 6 | 0.996(0.989-1.002) | 0-6 | 0.968(0.934-1.004) |
| 7 | 0.994(0.989-1.001) | 0-7 | 0.963(0.927-1.000) |
| 8 | 0.993(0.988-0.999) ^a^ | 0-8 | 0.956(0.919-0.995) ^a^ |
| 9 | 0.992(0.985-0.999) ^a^ | 0-9 | 0.949(0.911-0.988) ^a^ |
| 10 | 0.991(0.979-1.002) | 0-10 | 0.940(0.900-0.981) ^a^ |

RR: Relative risk; CI: confidence interval; a:*P* < 0.05.

**Table S6** The association between O_3_ exposure and SS hospitalizations in different subgroups (*RR*, 95% *CI*)

| lag | Male | Female | Age < 65 years | Age ≥ 65 years | Hot season | Cold season |
| --- | --- | --- | --- | --- | --- | --- |
| 0 | 0.995(0.912-1.086) | 0.991(0.967-1.015) | 0.992(0.964-1.020) | 0.989(0.949-1.030) | 0.991(0.969-1.014) | 0.965(0.934-0.997) ^a^ |
| 1 | 0.999(0.962-1.037) | 0.994(0.984-1.005) | 0.994(0.981-1.006) | 0.996(0.978-1.014) | 0.995(0.985-1.005) | 0.997(0.982-1.013) |
| 2 | 1.004(0.964-1.046) | 0.996(0.985-1.007) | 0.994(0.981-1.008) | 1.001(0.983-1.020) | 0.997(0.988-1.007) | 1.008(0.992-1.026) |
| 3 | 1.010(0.983-1.038) | 0.996(0.989-1.004) | 0.993(0.984-1.002) | 1.005(0.993-1.018) | 0.999(0.993-1.006) | 1.003(0.992-1.014) |
| 4 | 1.014(0.991-1.037) | 0.996(0.989-1.002) | 0.992(0.985-0.999) ^a^ | 1.007(0.996-1.018) | 1.000(0.994-1.006) | 0.996(0.988-1.005) |
| 5 | 1.014(0.988-1.041) | 0.995(0.988-1.002) | 0.991(0.983-1.000) | 1.007(0.995-1.019) | 1.000(0.994-1.006) | 0.993(0.983-1.003) |
| 6 | 1.012(0.987-1.039) | 0.994(0.987-1.002) | 0.991(0.983-0.999) ^a^ | 1.005(0.993-1.017) | 0.999(0.993-1.005) | 0.993(0.983-1.002) |
| 7 | 1.009(0.987-1.030) | 0.994(0.988-0.999) ^a^ | 0.991(0.984-0.998) ^a^ | 1.002(0.992-1.012) | 0.997(0.992-1.003) | 0.995(0.987-1.003) |
| 8 | 1.003(0.984-1.023) | 0.993(0.987-0.998) ^a^ | 0.991(0.985-0.998) ^a^ | 0.998(0.989-1.007) | 0.996(0.991-1.001) | 0.999(0.992-1.006) |
| 9 | 0.997(0.970-1.025) | 0.992(0.984-0.999) ^a^ | 0.992(0.983-1.001) | 0.993(0.981-1.006) | 0.993(0.987-1.001) | 1.004(0.994-1.014) |
| 10 | 0.990(0.948-1.034) | 0.991(0.979-1.003) | 0.992(0.978-1.006) | 0.988(0.969-1.008) | 0.991(0.981-1.001) | 1.010(0.994-1.026) |
| 0-0 | 0.995(0.912-1.086) | 0.991(0.967-1.015) | 0.992(0.964-1.020) | 0.989(0.949-1.030) | 0.991(0.969-1.014) | 0.965(0.934-0.997) |
| 0-1 | 0.994(0.892-1.108) | 0.985(0.956-1.015) | 0.985(0.951-1.021) | 0.985(0.935-1.037) | 0.986(0.958-1.015) | 0.962(0.924-1.002) |
| 0-2 | 0.998(0.891-1.118) | 0.981(0.951-1.032) | 0.980(0.944-1.017) | 0.986(0.934-1.041) | 0.983(0.954-1.015) | 0.970(0.928-1.015) |
| 0-3 | 1.008(0.896-1.136) | 0.978(0.945-1.011) | 0.973(0.935-1.012) | 0.991(0.936-1.049) | 0.983(0.952-1.015) | 0.974(0.928-1.022) |
| 0-4 | 1.022(0.905-1.154) | 0.974(0.940-1.008) | 0.965(0.927-1.005) | 0.998(0.941-1.058) | 0.983(0.951-1.016) | 0.970(0.922-1.020) |
| 0-5 | 1.037(0.914-1.175) | 0.969(0.935-1.004) | 0.957(0.918-0.998) ^a^ | 1.005(0.946-1.067) | 0.983(0.950-1.018) | 0.963(0.914-1.014) |
| 0-6 | 1.050(0.919-1.198) | 0.963(0.928-1.000) | 0.949(0.908-0.991) ^a^ | 1.010(0.948-1.075) | 0.982(0.947-1.018) | 0.956(0.905-1.010) |
| 0-7 | 1.058(0.920-1.217) | 0.957(0.920-0.996) ^a^ | 0.940(0.898-0.985) ^a^ | 1.012(0.947-1.081) | 0.980(0.943-1.018) | 0.951(0.898-1.008) |
| 0-8 | 1.062(0.918-1.228) | 0.950(0.912-0.990) ^a^ | 0.932(0.888-0.978) ^a^ | 1.009(0.942-1.081) | 0.976(0.938-1.015) | 0.950(0.893-1.010) |
| 0-9 | 1.059(0.912-1.230) | 0.943(0.904-0.983) ^a^ | 0.924(0.880-0.971) ^a^ | 1.003(0.934-1.076) | 0.969(0.931-1.010) | 0.954(0.894-1.018) |
| 0-10 | 1.049(0.894-1.230) | 0.934(0.893-0.977) ^a^ | 0.917(0.870-0.967) ^a^ | 0.991(0.919-1.069) | 0.961(0.920-1.003) | 0.963(0.898-1.034) |

*RR:* Relative risk; *CI:* confidence interval; a:*P* < 0.05.

**Table S7** The association between CO exposure and SS hospitalizations in Hefei from 2016 to 2021

| Single-day | *RR* (95% *CI*) | Multi-day | *RR* (95% *CI*) |
| --- | --- | --- | --- |
| 0 | 1.048(0.823-1.335) | 0-0 | 1.048(0.823-1.335) |
| 1 | 1.111(0.979-1.262) | 0-1 | 1.165(0.816-1.662) |
| 2 | 1.144(1.023-1.278) ^a^ | 0-2 | 1.332(0.895-1.983) |
| 3 | 1.127(0.994-1.277) | 0-3 | 1.500(0.977-2.306) |
| 4 | 1.071(0.956-1.199) | 0-4 | 1.607(1.006-2.566) ^a^ |
| 5 | 0.990(0.901-1.089) | 0-5 | 1.159(0.962-2.632) |
| 6 | 0.900(0.794-1.020) | 0-6 | 1.432(0.838-2.447) |
| 7 | 0.810(0.661-0.994) | 0-7 | 1.160(0.632-2.130) |

RR: Relative risk; CI: confidence interval; a:*P* < 0.05.

**Table S8** The association between CO exposure and SS hospitalizations in different subgroups (*RR*, 95% *CI*)

| lag | Male | Female | Age < 65 years | Age ≥ 65 years | Hot season | Cold season |
| --- | --- | --- | --- | --- | --- | --- |
| 0 | 0.554(0.180-1.708) | 1.071(0.837-1.370) | 1.081(0.810-1.443) | 0.962(0.621-1.491) | 1.284(0.878-1.878) | 0.989(0.800-1.222) |
| 1 | 0.657(0.356-1.213) | 1.131(0.994-1.287) | 1.107(0.951-1.289) | 1.116(0.890-1.401) | 1.166(0.951-1.430) | 1.090(0.977-1.217) |
| 2 | 0.758(0.461-1.247) | 1.160(1.035-1.299) ^a^ | 1.110(0.971-1.269) | 1.227(1.007-1.496) ^a^ | 1.056(0.892-1.252) | 1.157(1.051-1.274) ^a^ |
| 3 | 0.837(0.486-1.442) | 1.138(1.001-1.294) ^a^ | 1.081(0.930-1.256) | 1.274(0.997-1.561) | 0.954(0.791-1.151) | 1.160(1.040-1.293) ^a^ |
| 4 | 0.892(0.542-1.470) | 1.077(0.959-1.209) | 1.026(0.896-1.175) | 1.188(0.970-1.454) | 0.859(0.724-1.020) | 1.110(1.006-1.224) ^a^ |
| 5 | 0.926(0.604-1.419) | 0.991(0.900-1.092) | 0.955(0.852-1.070) | 1.077(0.910-1.275) | 0.772(0.666-0.895) | 1.025(0.945-1.112) ^a^ |
| 6 | 0.944(0.552-1.617) | 0.891(0.789-1.018) | 0.879(0.757-1.021) | 0.945(0.755-1.183) | 0.693(0.573-0.837) | 0.925(0.830-1.031) |
| 7 | 0.954(0.407-2.237) | 0.803(0.651-0.989) | 0.803(0.630-1.025) | 0.816(0.565-1.180) | 0.621(0.460-0.840) | 0.825(0.690-0.987) |
| 0-0 | 0.554(0.180-1.708) | 1.071(0.837-1.370) | 1.081(0.810-1.443) | 0.962(0.621-1.491) | 1.284(0.878-1.878) | 0.989(0.801-1.222) |
| 0-1 | 0.364(0.067-1.968) | 1.211(0.844-1.738) | 1.197(0.784-1.829) | 1.074(0.566-2.039) | 1.497(0.850-2.636) | 1.078(0.791-1.470) |
| 0-2 | 0.276(0.041-1.881) | 1.404(0.938-2.103) | 1.329(0.827-2.138) | 1.318(0.646-2.690) | 1.581(0.834-2.998) | 1.247(0.882-1.763) |
| 0-3 | 0.231(0.029-1.839) | 1.598(1.033-2.472) ^a^ | 1.437(0.859-2.402) | 1.644(0.765-3.534) | 1.509(0.758-3.004) | 1.446(0.996-2.101) |
| 0-4 | 0.206(0.022-1.948) | 1.721(1.070-2.768) ^a^ | 1.473(0.841-2.581) | 1.952(0.850-4.484) | 1.296(0.616-2.728) | 1.605(1.069-2.411) ^a^ |
| 0-5 | 0.191(0.017-2.133) | 1.706(1.023-2.844) ^a^ | 1.407(0.770-2.572) | 2.102(0.860-5.138) | 1.001(0.452-2.218) | 1.646(1.062-2.551) ^a^ |
| 0-6 | 0.180(0.014-2.398) | 1.528(0.886-2.635) | 1.237(0.651-2.350) | 1.987(0.765-5.163) | 0.693(0.297-1.619) | 1.523(0.952-2.435) ^a^ |
| 0-7 | 0.172(0.009-3.156) | 1.227(0.661-2.277) | 0.993(0.480-2.054) | 1.623(0.546-4.821) | 0.431(0.166-1.120) | 1.257(0.735-2.148) ^a^ |

*RR:* Relative risk; *CI:* confidence interval; a:*P* < 0.05.


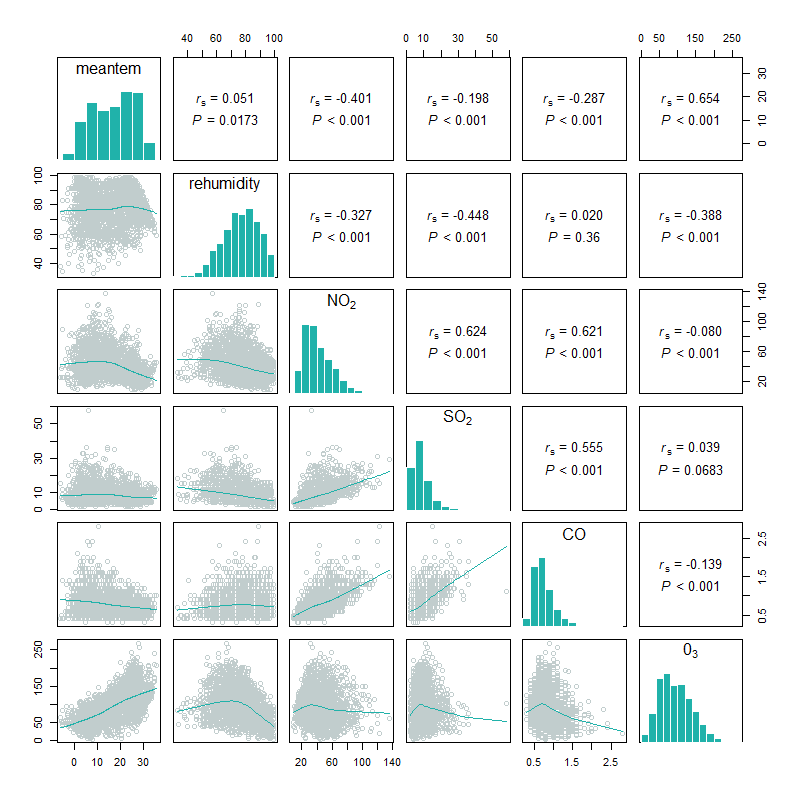


**Figure S1** The spearman's correlation coefficients between different meteorological factors and gaseous pollutants


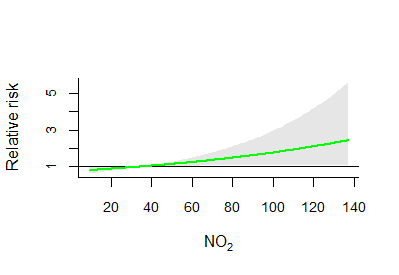

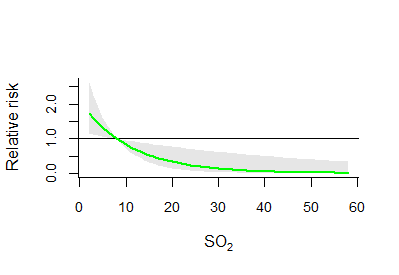


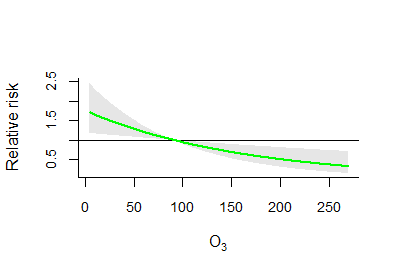

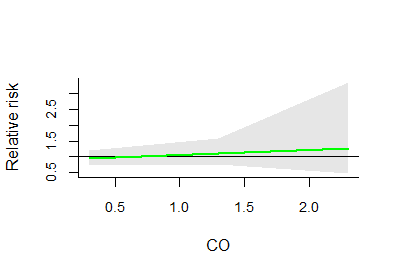


**Figure S2** Concentration-response curves of daily hospitalizations for SS and NO_2_, SO_2_, O_3_,

and CO levels


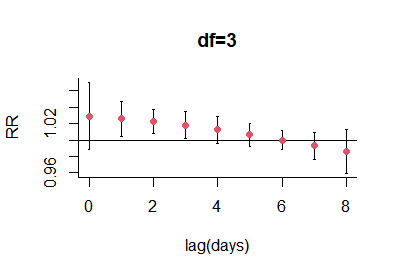

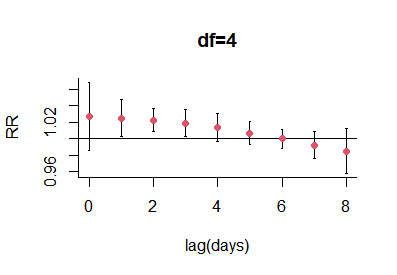

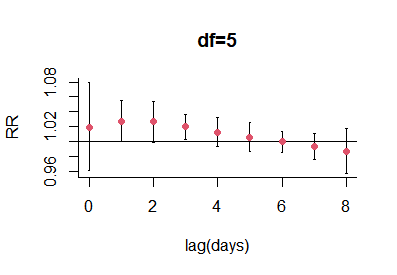


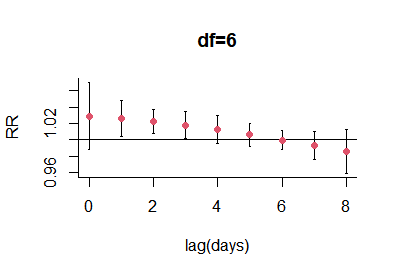

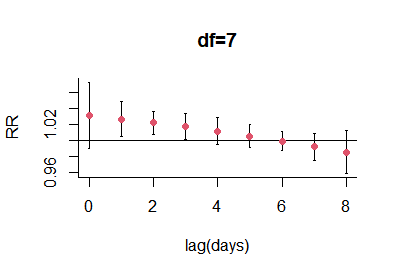

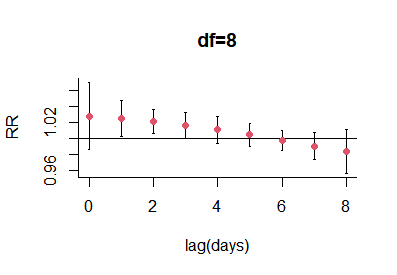


**Figure S3** The single-day association between NO_2_ exposure and SS hospitalizations when varying the degrees of freedom (3-5 *dfs*) for SO_2_, relative humidity and the df (6-8 *dfs*/year) for time


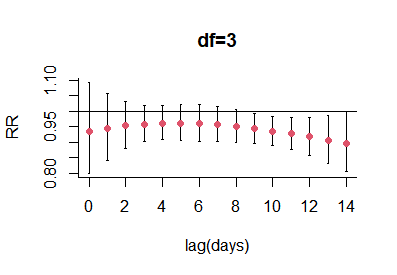

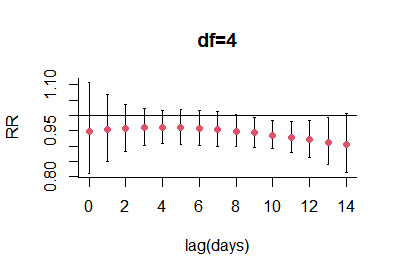

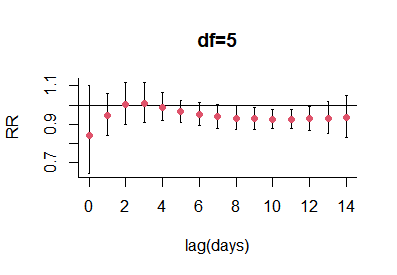

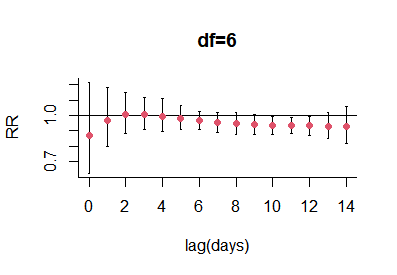

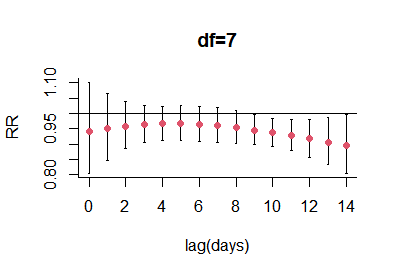

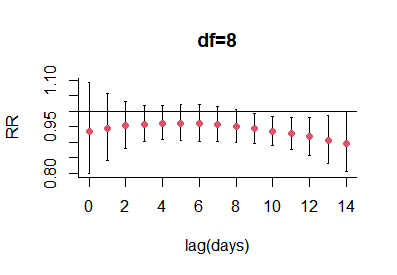


**Figure S4** The single-day association between SO_2_ exposure and SS hospitalizations when varying the degrees of freedom (3-5 *dfs*) for SO_2_, relative humidity and the df (6-8 *dfs*/year) for time


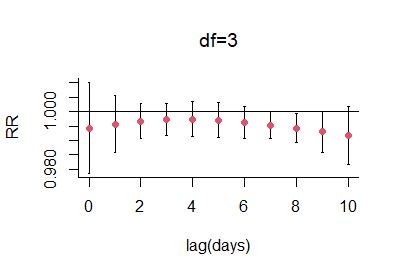

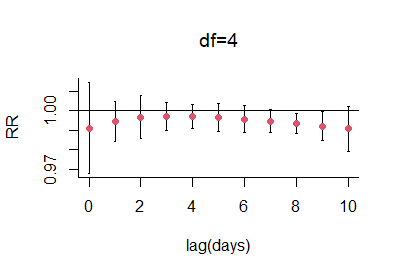

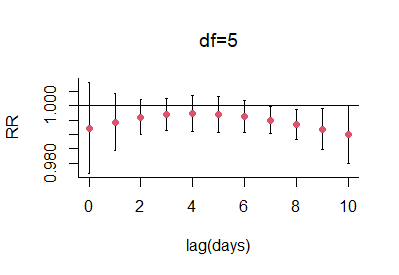

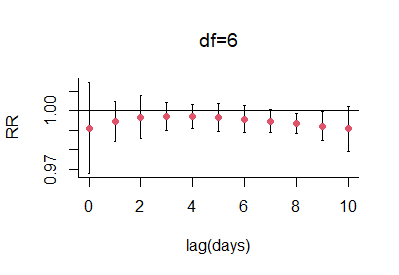

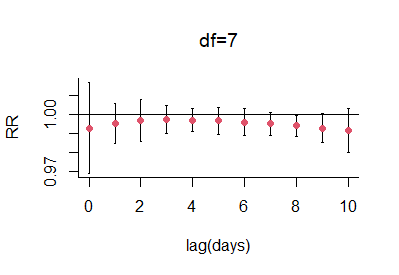

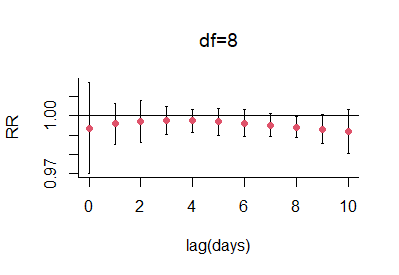


**Figure S5** The single-day association between O_3_ exposure and SS hospitalizations when varying the degrees of freedom (3-5 *dfs*) for SO_2_, relative humidity and the df (6-8 *dfs*/year) for time


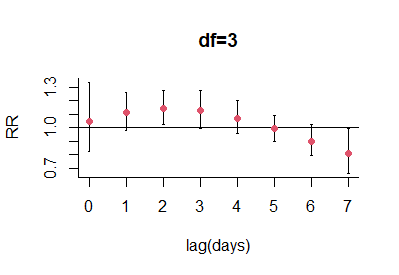

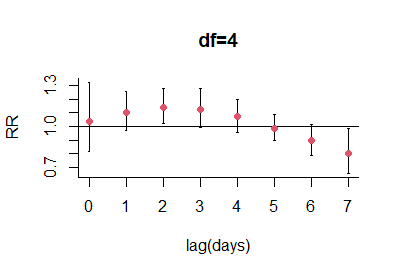

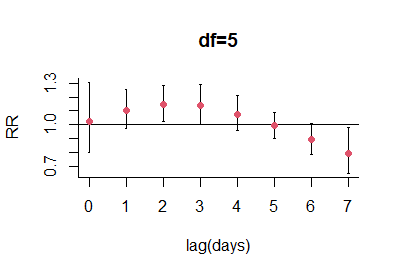

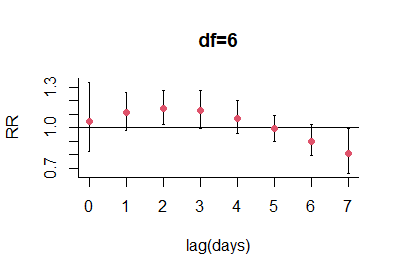

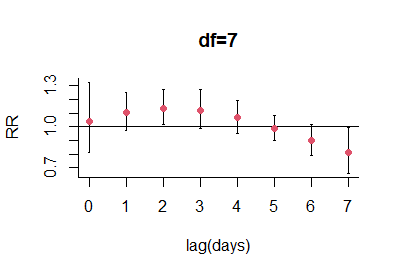

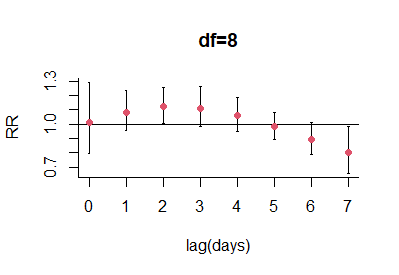


**Figure S6** The single-day association between CO exposure and SS hospitalizations when varying the degrees of freedom (3-5 *dfs*) for SO_2_, relative humidity and the df (6-8 *dfs*/year) for time
